# Heritability of Behavioral Problems in 7-Year Olds Based on Shared and Unique Aspects of Parental Views

**DOI:** 10.1007/s10519-016-9823-1

**Published:** 2016-10-28

**Authors:** Iryna O. Fedko, Laura W. Wesseldijk, Michel G. Nivard, Jouke-Jan Hottenga, Catharina E. M. van Beijsterveldt, Christel M. Middeldorp, Meike Bartels, Dorret I. Boomsma

**Affiliations:** 1 Department of Biological Psychology, Vrije Universiteit Amsterdam, Van der Boechorststraat 1, 1081BT Amsterdam, The Netherlands; 2 EMGO+ Institute for Health and Care Research, VU Medical Center, Amsterdam, The Netherlands; 3 Neuroscience Campus Amsterdam, Amsterdam, The Netherlands; 4 Department of Child and Adolescent Psychiatry, GGZ inGeest/VU Medical Center, Amsterdam, The Netherlands

**Keywords:** CBCL 6–18, Parental ratings, Heritability, Genetic correlation, Behavioral problems

## Abstract

In studies of child psychopathology, phenotypes of interest are often obtained by parental ratings. When behavioral ratings are obtained in the context of a twin study, this allows for the decomposition of the phenotypic variance, into a genetic and a non-genetic part. If a phenotype is assessed by a single rater, heritability is based on the child’s behavior as expressed in the presence of that particular rater, whereas heritability based on assessments by multiple raters allows for the estimation of the heritability of the phenotype based on rater agreement, as well as the heritability of the rater specific view of the behavior. The aim of this twin study was to quantify the rater common and rater specific contributions to the variation in children’s behavioral problems. We estimated the heritability of maternal and paternal ratings of the Child Behavior Checklist (CBCL) 6–18 empirical emotional and behavioral problem scales in a large sample of 12,310 7-year old Dutch twin pairs. Between 30 and 59% of variation in the part of the phenotype parents agree upon was explained by genetic effects. Common environmental effects that make children in the same family similar explained less variance, ranging between 0 and 32%. For unique views of their children’s behavioral problems, heritability ranged between 0 and 20% for maternal and between 0 and 22% for paternal views. Between 7 and 24% of the variance was accounted for by common environmental factors specific to mother and father’s views. The proportion of rater shared and rater specific heritability can be translated into genetic correlations between parental views and inform the design and interpretation of results of molecular genetic studies. Genetic correlations were nearly or above 0.7 for all CBCL based psychopathology scales. Such large genetic correlations suggest two practical guidelines for genome-wide association studies (GWAS): when studies have collected data from either fathers or mothers, the shared genetic aetiology in parental ratings indicates that is possible to analyze paternal and maternal assessments in a single GWAS or meta-analysis. Secondly, if a study has collected information from both parents, a gain in statistical power may be realized in GWAS by the simultaneous analysis of the data.

**Electronic supplementary material:**

The online version of this article (doi:10.1007/s10519-016-9823-1) contains supplementary material, which is available to authorized users.

## Introduction

To assess children’s behavioral and emotional problems, researchers often rely on parental ratings. However, parents are not always in agreement on the behavior of their child. Maternal and paternal ratings on the Child Behavior Checklist (CBCL) 6–18, for example, correlate around 0.75, which is lower than the average test–retest reliability of the instrument, which is 0.89 for the empirical subscales (Achenbach and Rescorla 2001; Achenbach et al. 2003; Achenbach and Ruffle 2000). Differences in parental normative standards or perception of child’s behavior could explain why the correlations between parents are below the test retest reliability; an alternative or additional explanation involves the existence of specific parental views on the child’s behaviors if a child behaves differently in the presence of each parent (Bartels et al. 2007a; Hewitt et al. 1992; Kan et al. 2014). Maternal ratings are the most common single informant assessment found in the literature. However, as children interact with both parents, adding paternal observations may provide additional information about a child’s behavior.

The Child Behavior Checklist 6–18 (CBCL 6–18) assesses child behavioral and emotional problems on a number of scales that indicate problems in the internalizing (INT) domain (anxious/depressed, withdrawn/depressed, somatic complaints) and externalizing (EXT) domain (rule-breaking, and aggressive behavior) as well as social, thought, attention problems, dysregulation, which sums anxious/depressed, aggressive behavior and attention problems (Althoff et al. 2010), and total problems. The contribution of genetic (twin heritability) and environmental effects to the variation in rater agreement and disagreement of some of these scales were explored for children of age 7 years and showed that the common part of multi-informant assessments was the most heritable, ranging from 24 to 51% (Abdellaoui et al. 2008; Bartels et al. 2007a; Boomsma et al. 2005; Hoekstra et al. 2008; Van der Valk et al. 2003), free of possible rater bias and specific parental views. Specific parental views usually were less heritable, ranging from 4 to 24% across the studies, scales and domains, but still provided information about child behavior. Phenotypes such as somatic complaints, rule-breaking behavior, social problems and the dysregulation profile received less attention.

In molecular genetic studies, heritability as estimated in the twin model is often contrasted with SNP-heritability, the phenotypic variance explained by a large subset of all common genetic variants (single nucleotyde polymorphisms, SNPs). SNP-heritability can be obtained from genomic-relatedness-matrix restricted maximum likelihood (GREML) analysis (Benjamin et al. 2012; Yang et al. 2011) where the effect of individual genetic variants can be estimated in genome wide association studies (GWAS). In general, SNP heritability and twin-heritability are correlated across traits, i.e. traits with high twin heritability tend to have a high SNP-heritability. The power to detect genetic variants in a GWAS in turn is also related to, among other factors, the SNP and twin heritability estimates. If child behavioral problems assessed by multiple informants, for example mother, father or teacher are more heritable, due to the focus on the part of the behavior on which all raters agree and with reduction of measurement error, rater bias or specific rater view, power will be increased in a GWAS by combining information from different raters. Alternatively, a substantial rater specific heritability might indicate that ratings from particular informants should be analyzed separately in GWAS, to identify variants contributing to that part of the behavior that is only seen by a specific rater in a specific context. Results obtained from twin studies with multiple informants may address these questions and convey additional information, which can aid in the design of molecular genetic studies and results interpretation.

The aim of this study was to estimate the relative contribution of genetic factors (twin heritability) to the raters agreement and disagreement of the all empirical scales of CBCL 6–18 in a large sample (N = 12,310 pairs) of twins around age 7 years and in this way inform molecular studies. These twins participate in an ongoing longitudinal data collection for the Netherlands Twin Register (Boomsma et al. 2006; van Beijsterveldt et al. 2013). We investigated agreement and disagreement between parents in the psychometric model (Bartels et al. 2007a; Hewitt et al. 1992). The large sample size allowed us to exploit the liability threshold model (Falconer et al. 1996) and consider data as categorical, as in population based samples CBCL scales tend to be skewed. It has been shown that this approach has an advantage over various data transformations for skewed data (Derks et al. 2004). The large sample further allowed for the assessment of quantitative and qualitative sex differences.

## Methods

### Participants and measures

The data analyzed in this study are obtained by the Netherlands Twin Register (NTR), which is a population-based longitudinal study of the health and life style of twins and their families. Participants are voluntarily registered with the NTR and the data collection protocol was approved by the Medical Research Ethics Committee of the VU University Medical Center. For 12,629 twin pairs, born between 1986 and 2006, maternal and paternal ratings were available. Data from 312 pairs were excluded since one or both twins had an illness or handicap that interfered with daily functioning. For the same-sex twin pairs zygosity was determined by blood group (n pairs = 194), DNA polymorphisms (n pairs = 1558) or by parental zygosity questionnaire (n pairs = 6661). Twins for whom zygosity was unknown (n pairs = 7) were also excluded from the analysis. The final sample comprised 12,310 twin pairs: 2079 monozygotic male (MZM), 2086 dizygotic male (DZM), 2324 monozygotic female (MZF), 1924 dizygotic female (DZF) and 3897 opposite-sex pairs (DOS). CBCL data were collected when the twin pair was about 7 years old (mean = 7.45, SD = 0.40, N = 24,620). Maternal questionnaires were available for 12,086 pairs, paternal questionnaires for 8555 pairs. Either the CBCL 4-18 (Achenbach 1991) or the CBCL 6–18 (Achenbach and Rescorla 2001) were used, depending on the year in which the questionnaire was sent to participants. The sum scores for each scale were computed based on syndrome scale (version Achenbach and Rescorla 2001). Means, standard deviations, and information on skewness and kurtosis for all scales are provided in Supplementary Table 1. The scale scores are the sum of all items, where a lower score indicates less or no behavior problems and higher scores indicate the presence of behavioral problems. Because twin studies represent population samples, the distribution of CBCL data is often skewed (L-shaped). This could lead to biased parameter estimates (Derks et al. 2004). Therefore, we categorized the data into 3 categories (0, 1, 2) and carried out the analyses using a liability threshold model. The two thresholds approximately divided the dataset with both parental ratings into 3 equal parts. The liability threshold model assumes an underlying normal distribution, which we scaled with a mean of 0 and unit variance. In this context, thresholds reflect the prevalences of childhood psychopathology rated by mother and father. Descriptive statistics were calculated with SPSS version 21 (SPSS 2012). Relationships between raw data and categories can be found in Supplementary Table 2.

### Genetic epidemiological analyses

For each CBCL scale, a 4 × 4 polychoric correlation matrix was estimated in all zygosity by sex groups (MZM, DZM, MZF, DZF and DOS). It contained parental twin1-twin2 correlations, the parental cross-correlations between twins (e.g. father rating of twin1 and mother rating of twin2) and the parental agreement correlations (Table 1). We constrained the correlations, such that (1) parental agreement correlations across sex and zygosity were equal, and (2) parental twin1–twin2 correlations across sex within MZ and DZ pairs were equal. The most parsimonious models, in terms of the constraints outlined above, were used in subsequent genetic analyses. A psychometric genetic model, as described by Hewitt et al. (1992) and Bartels et al. (2007a) was fitted to the data to estimate heritability and to disentangle shared and specific aspects of the parental ratings of the child’s behavior. The model specifies a common component to the phenotype, as assessed by both parents and a unique component of the child’s phenotype reflected in the assessments of each parent. The total variance of mother’s ratings (Vmother) is decomposed into common (Vshared) and unique (Vunique, mother) parts. The total variance of father’s rating (Vfather) is decomposed in the same way. Vshared is decomposed into variance components representing additive genetic (Va, shared), common environment (Vc, shared) or dominant genetic (Vd, shared), and unique environment (Ve, shared) components. The additive genetic variance (Va, shared) represents the part of the heritability of the phenotype that is assessed by both parents. Likewise (Vunique, mother) is decomposed into (Va, unique, mother), (Vc, unique, mother) or (Vd, unique, mother), and (Ve, unique, mother). Vunique, father is decomposed in the same way. The additive genetic variance of the unique component of mother or father ratings (Va, unique) represents the part of the heritability of the trait that is uniquely expressed in the presence of each parent. Whether parents truly rated the specific aspect of the child behavior was tested by constraining the genetic variance of the specific view (Va, unique) to 0. We also tested if common environmental variance of the shared aspect of the phenotype (Vc, shared), which is free of bias and specific parental view, equaled 0. The rater bias is reflected in the proportion of common environmental variance of raters disagreement (Vc, unique). The genetic correlation between maternal and paternal ratings was computed based on the estimates of the additive genetic components of the most parsimonious model based on the formula: $r_g\, = \,Va, \,shared/\left( {\sqrt {Va, \,shared\, + \,Va,\,unique,\,mother} \, \times \,\sqrt {Va,\, shared\, + \,Va,\,unique,\,father} } \right).$ The level of significance was 0.05/12 = 0.0042 to account for multiple testing of 12 CBCL scales. Analyses were performed in OpenMx 2.2.6 (Neale et al. 2015).

**Table 1:** 4 × 4 correlation matrix for 5 zygosity by sex groups

	Mother twin 1	Father twin 1	Mother twin 2	Father twin 2
Mother twin 1	1	Parental agreement correlation	Mother correlation twin1–twin2	Mother (twin1)–father (twin2) cross-correlation
Father twin 1	Parental agreement correlation	1	Father (twin1)–mother (twin2) cross-correlation	Father correlation twin1–twin2
Mother twin 2	Mother correlation twin1–twin2	Father (twin1)–mother (twin2) cross-correlation	1	Parental agreement correlation
Father twin 2	Mother (twin1)–father (twin2) cross-correlation	Father correlation twin1–twin2	Parental agreement correlation	1

## Results

### Descriptive statistics

Means and standard deviations for boys and girls for mother and father ratings are given in Table 2, which also gives the thresholds. For all CBCL scales, the means of the sum scores were higher for maternal than for paternal ratings and ratings for boys and girls were significantly different. Both mothers and fathers rated girls higher for the anxious/depressed and somatic complaints subscales and the internalizing scale. For all other scales boys scored higher than girls, with the exception of the withdrawn/depressed scale, for which they scored similarly. Differences in prevalences between boys and girls were reflected in the significant loss of fit of the model (Supplementary Material, Table 3) when constraining the thresholds to be the same across sexes.

**Table 2:** Means, and standard deviations of the untransformed data and thresholds estimated for categorical transformation of data

	Anxious/depressed		Withdrawn/depressed		Somatic complaints		Rule-breaking behavior		Aggressive behavior		Social problems		Thought problems		Attention problems		INT		EXT		Dysregulation profile		Total problems	
	Mo	Fa	Mo	Fa	Mo	Fa	Mo	Fa	Mo	Fa	Mo	Fa	Mo	Fa	Mo	Fa	Mo	Fa	Mo	Fa	Mo	Fa	Mo	Fa
Boys																								
Mean	2.19	1.68	1.19	0.96	1.13	0.83	1.59	1.37	5.87	5.03	2.28	1.86	1.71	1.33	3.58	3.16	4.49	3.47	7.45	6.40	11.63	9.86	23.11	19.13
SD	2.58	2.12	1.68	1.51	1.60	1.30	2.04	1.87	5.37	4.83	2.59	2.29	2.19	1.86	3.27	3.02	4.61	3.86	6.92	6.23	9.28	8.30	17.70	15.56
Threshold 1	0.06	0.27	−0.11	0.07	−0.05	0.16	−0.24	−0.14	−0.43	−0.29	0.02	0.17	−0.34	−0.17	−0.34	−0.25	−0.19	0.06	−0.56	−0.43	−0.53	−0.34	−0.56	−0.34
Threshold 2	0.48	0.70	0.59	0.75	0.59	0.84	0.37	0.45	0.37	0.52	0.44	0.62	0.27	0.47	0.49	0.59	0.53	0.81	0.29	0.44	0.32	0.50	0.23	0.45
Girls																								
Mean	2.36	1.80	1.14	0.92	1.29	0.92	1.12	0.97	4.48	3.89	1.98	1.66	1.32	0.94	2.62	2.32	4.78	3.63	5.59	4.86	9.47	8.01	19.41	15.88
SD	2.61	2.19	1.59	1.42	1.74	1.39	1.61	1.49	4.42	4.03	2.29	2.02	1.83	1.50	2.83	2.65	4.72	3.92	5.60	5.07	8.02	7.25	15.69	13.84
Threshold 1	−0.03	0.19	−0.10	0.08	−0.15	0.09	−0.02	0.07	−0.19	−0.06	0.11	0.27	−0.14	0.09	0.01	0.10	−0.26	−0.01	−0.33	−0.21	−0.28	−0.10	−0.33	−0.09
Threshold 2	0.39	0.63	0.64	0.80	0.48	0.73	0.62	0.71	0.66	0.79	0.57	0.72	0.49	0.74	0.83	0.91	0.47	0.78	0.59	0.70	0.62	0.80	0.49	0.71

### Correlations between twins and raters

Correlations, estimated in 4 × 4 matrix for each of the five zygosity by sex groups are summarized in Table 3. For all scales parental agreement correlations were similar between boys and girls as well as between MZ and DZ twins. Parental agreement correlations were constrained to be equal across sex and zygosity, this did not lead to significant worsening of fit of the model to the data (Supplementary Table 3). We detected sex effects for the aggressive behavior, externalizing scale and dysregulation profile reflected by significant sex differences in parental agreement, but since the differences were small, we decided not to model sex specific effects in the variance decomposition. Parental twin correlations and cross-twin-cross-rater-correlations were higher for MZ twins, than for DZ twins, indicating that rater disagreement partly reflects a rater specific or context specific view and not only rater bias. Parental twin correlations were similar for boys and girls within MZ and DZ pairs, and therefore were constrained to be the equal across sex in subsequent submodels (Supplementary Table 3).

**Table 3:** Correlations estimated from a saturated model: parental agreement, twin correlations and twin cross-correlations

	Anxious/depressed		Withdrawn/depressed		Somatic complaints		Rule-breaking behavior		Aggressive behavior		Social problems		Thought problems		Attention problems		INT		EXT		Dysregulation profile		Total problems	
Parental agreement^a^																								
MZM	0.65		0.61		0.68		0.65		0.75		0.67		0.63		0.74		0.65		0.75		0.75		0.74	
DZM	0.66		0.62		0.65		0.64		0.74		0.69		0.64		0.74		0.68		0.74		0.75		0.74	
MZF	0.67		0.61		0.66		0.60		0.70		0.64		0.63		0.73		0.65		0.70		0.71		0.71	
DZF	0.64		0.62		0.66		0.62		0.70		0.66		0.64		0.73		0.63		0.69		0.68		0.71	
Twin correlations for mother and father ratings																								
	Mo	Fa	Mo	Fa	Mo	Fa	Mo	Fa	Mo	Fa	Mo	Fa	Mo	Fa	Mo	Fa	Mo	Fa	Mo	Fa	Mo	Fa	Mo	Fa
MZM	0.71	0.74	0.72	0.74	0.69	0.71	0.90	0.91	0.88	0.90	0.80	0.82	0.78	0.83	0.81	0.81	0.74	0.76	0.89	0.89	0.87	0.88	0.90	0.90
DZM	0.42	0.43	0.34	0.31	0.44	0.43	0.68	0.69	0.60	0.62	0.52	0.54	0.48	0.48	0.29	0.32	0.51	0.48	0.63	0.64	0.62	0.64	0.69	0.71
MZF	0.74	0.76	0.74	0.76	0.72	0.70	0.88	0.89	0.86	0.88	0.78	0.83	0.80	0.81	0.77	0.81	0.77	0.77	0.89	0.88	0.87	0.88	0.89	0.90
DZF	0.46	0.50	0.34	0.46	0.45	0.41	0.70	0.73	0.58	0.63	0.50	0.54	0.53	0.52	0.24	0.26	0.55	0.57	0.62	0.70	0.62	0.66	0.71	0.77
DOS	0.49	0.47	0.41	0.44	0.46	0.43	0.68	0.67	0.57	0.59	0.48	0.54	0.46	0.49	0.30	0.36	0.57	0.55	0.62	0.63	0.58	0.63	0.68	0.70
Mother—father cross-correlations																								
MZM	0.45		0.44		0.46		0.60		0.67		0.54		0.51		0.61		0.49		0.67		0.66		0.67	
DZM	0.22		0.12		0.23		0.42		0.44		0.34		0.29		0.17		0.30		0.46		0.45		0.50	
MZF	0.50		0.46		0.45		0.52		0.61		0.51		0.51		0.57		0.51		0.62		0.63		0.64	
DZF	0.26		0.20		0.22		0.43		0.39		0.33		0.34		0.11		0.32		0.44		0.40		0.52	
DOS	0.28		0.21		0.26		0.40		0.38		0.31		0.29		0.20		0.33		0.41		0.40		0.47	

^a^In DOS twin pairs scores were organized in such a way, that boys are first and girls are second. Therefore in 4 × 4 matrix parental agreement for twin 1 is a correlation for a boy and parental agreement for twin 2 is a correlation for a girl and DZM and DZF correlations reflect DOS correlations

Table 4 summarizes the correlations obtained from the constrained model. For all scales, except attention problems, parental correlations in MZ twin pairs were lower than one and twice the DZ correlations, or less, indicating contributions of Additive Genetic (V_A_), Shared Environmental (V_C_) and Unique Environmental (V_E_) variation to the total phenotypic variation. For attention problems MZ correlations were lower than one and larger than twice the DZ correlations, indicating Additive Genetic (V_A_), Dominant Genetic (V_D_) and Unique Environmental (V_E_) variation. Thus, for all traits a V_ACE_ variance decomposition model was fitted, except for attention problems, for which an V_ADE_ model was used.

**Table 4:** Correlations estimated from the most parsimonious model

	Zygosity	Twin correlation		Mother–father cross-correlations	Parental agreement	
		Mo	Fa		Phenotypic correlation	Genetic correlation
Anxious/depressed	MZ	0.73	0.75	0.48	0.66	0.87
	DZ	0.46	0.47	0.26		
Withdrawn/depressed	MZ	0.73	0.75	0.46	0.62	0.72
	DZ	0.37	0.41	0.18		
Somatic complaints	MZ	0.70	0.71	0.45	0.66	0.78
	DZ	0.45	0.42	0.24		
Rule-breaking behavior	MZ	0.89	0.90	0.56	0.63	0.74
	DZ	0.69	0.69	0.41		
Aggressive behavior	MZ	0.87	0.89	0.64	0.72	0.86
	DZ	0.58	0.61	0.40		
Social problems	MZ	0.79	0.83	0.54	0.67	0.77
	DZ	0.50	0.54	0.32		
Thought problems	MZ	0.79	0.82	0.52	0.64	0.68
	DZ	0.48	0.49	0.30		
Attention problems	MZ	0.79	0.81	0.59	0.74	0.42/1.0/0.74^a^
	DZ	0.29	0.32	0.17		
INT	MZ	0.75	0.77	0.51	0.65	0.89
	DZ	0.55	0.53	0.32		
EXT	MZ	0.89	0.89	0.64	0.72	0.82
	DZ	0.62	0.65	0.43		
Dysregulation profile	MZ	0.87	0.88	0.64	0.72	0.89
	DZ	0.60	0.64	0.42		
Total problems	MZ	0.89	0.90	0.66	0.73	0.90
	DZ	0.69	0.72	0.49		

^a^For attention problems both the additive genetic correlation, the correlation between genetic dominance factors and the total genetic correlation (the correlation between the summed additive and dominant genetic effects) are given

### Genetic psychometric model

**Fig. 1:**
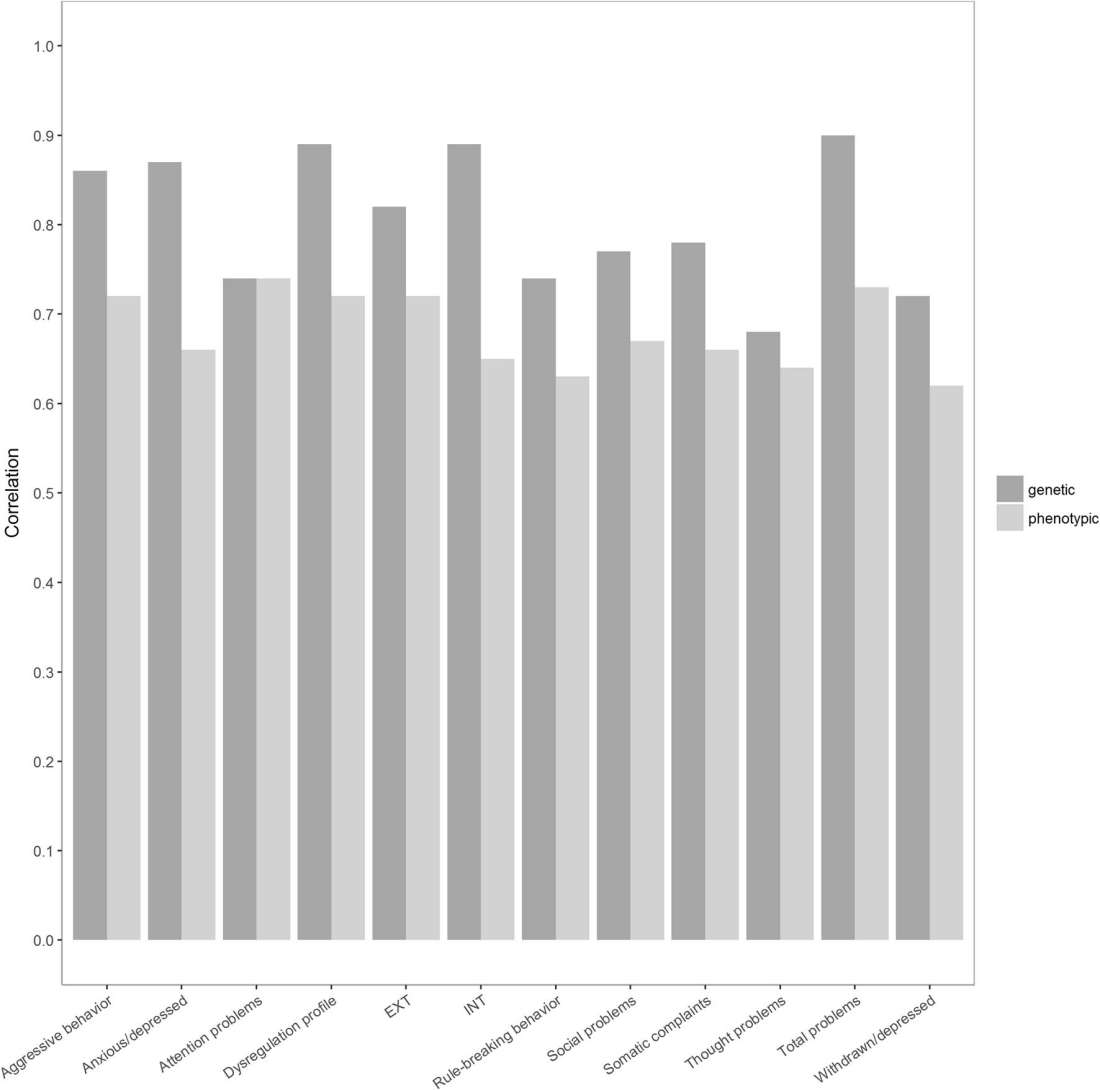
Genetic and phenotypic correlations between maternal and paternal ratings across all CBCL 6–18 scales. For all scales, except attention problems, the genetic correlations between additive genetic factors are depicted. For attention problems scale the *total* genetic correlation (between summed additive and dominant effects) is shown

For all scales, the common component of the phenotype assessed by both parents was substantial, with parental ratings correlations varying between 0.62 and 0.74 (Fig. 1; Table 4); however, a contribution of specific aspects of the child’s phenotype was present as well. For all scales, a substantial amount of the total variance ranging from 34 to 65% (Table 5) was accounted for by additive genetic variation, of which 15–48% was shared between parents (agreement) and 0–22% was unique to each parent. For all scales, except attention problems, between 7 and 56% of total variation was accounted for by common environmental factors. The proportion of such factors that contribute to variation in parental agreement, i.e. free of rater bias and specific parental view, ranged from 0 to 32%. The proportion that is unique to each parent’s perspective ranged from 7 to 24%. The contribution of dominant genetic effects to the total variation in the attention problems was 44% and was reflected in the part of the phenotype that parents agreed upon. Genetic correlations, computed based on additive genetic components of the most parsimonious psychometric model, ranged from 0.42 to 0.90 (Fig. 1; Table 4). The additive genetic correlation of 0.42 is an outlier, which was observed for attention problems. This is the only scale for which dominant effects were found and the dominance genetic correlation was one. The total genetic correlation (the correlation between the summed additive and dominant genetic effects) was 0.74.

**Table 5:** Heritability (A), shared (C) and unique (E) environmental effects, estimated from the most parsimonious psychometric model for each of the empirical scale of CBCL 6–18

	Phenotype, parents agree upon (%)	Unique mother’s assessment of the phenotype (%)	Unique father’s assessment of the phenotype (%)	Total mother’s assessment (%)	Total father’s assessment (%)
Anxious/depressed					
A	48 (46–50)	–	16 (11–21)	48	64
C	–	23 (21–25)	12 (10–16)	23	12
E	17 (15–19)	12 (10–13)	7 (5–9)	29	24
Withdrawn/depressed					
A	45 (43–47)	20 (15–26)	15 (12–21)	65	60
C	–	7 (3–13)	15 (10–20)	7	15
E	17 (16–19)	11 (9–12)	8 (7–10)	28	25
Somatic complaints					
A	45 (42–47)	9 (3–14)	16 (12–22)	54	61
C	–	17 (13–21)	10 (6–16)	17	10
E	21 (19–23)	8 (7–10)	8 (6–10)	29	29
Rule-breaking behavior					
A	30 (27–34)	11 (10–14)	10 (7–14)	41	40
C	26 (22–29)	22 (19–25)	23 (20–27)	48	49
E	7 (6–8)	4 (3–5)	4 (3–5)	11	11
Aggressive behavior					
A	48 (44–52)	9 (6–13)	7 (5–11)	57	55
C	16 (12–20)	14 (10–17)	17 (14–21)	30	33
E	8 (7–9)	5 (4–6)	3 (2–4)	13	11
Social problems					
A	45 (40–51)	14 (9–20)	13 (7–18)	59	58
C	9 (5–14)	11 (6–15)	16 (11–20)	20	25
E	13 (11–14)	8 (6–10)	5 (3–6)	21	18
Thought problems					
A	43 (38–48)	18 (17–24)	22 (17–25)	61	65
C	8 (4–12)	9 (7–14)	8 (4–13)	17	16
E	12 (11–13)	9 (7–11)	6 (5–8)	21	18
Attention problems					
A	15 (9–24)	20 (19–22)	22 (21–24)	35	37
D	44 (36–50)	–	–	44	44
E	15 (14–17)	6 (4–7)	4 (2–5)	21	19
INT					
A	39 (34–43)	–	10 (5–15)	39	49
C	12 (8–16)	24 (22–26)	16 (12–21)	36	28
E	15 (13–16)	11 (9–12)	8 (7–10)	26	23
EXT					
A	41 (38–45)	12 (8–15)	6 (4–10)	53	47
C	22 (19–26)	13 (10–16)	19 (15–22)	35	41
E	8 (7–9)	3 (2–4)	3 (3–4)	11	11
Dysregulation profile					
A	45 (41–49)	12 (9–16)	–	57	45
C	19 (16–23)	11 (8–15)	23 (22–25)	30	42
E	8 (7–9)	4 (3–6)	5 (4–6)	12	13
Total problems					
A	34 (31–38)	8 (5–11)	–	42	34
C	32 (28–35)	16 (13–19)	24 (22–25)	48	56
E	7 (6–8)	3 (2–4)	3 (3–4)	10	10

## Discussion

In this study we employed a psychometric model to determine to what extent parental assessments of a child’s behavioral problems around age 7 reflect common and parent specific aspects of the child behavior or if parents disagree due to rater bias. We observed interparental phenotypic correlations between 0.62 and 0.74, reflecting substantial but incomplete agreement between parents. Incomplete agreement may result in different heritability estimates between a single phenotype as assessed by different informants. Different informants provide information about child’s behavior and it is important to identify, prior to large GWAS efforts, whether the additive genetic effects on a trait strictly are found in the phenotypic variation which correlates between raters. Our analyses showed these were fairly highly correlated and that genetic correlations ranged from moderate to high (Fig. 1; Table 4), that is from 0.68 to 0.90 for all problem scales, with the exception of attention problems. The attention problems scale, as observed in numerous studies, has a different genetic architecture with non-additive genetic influences explaining a substantial part of the heritability.

### Comparison to previous results

In the NTR exploration of parental rater bias effect were conducted earlier for anxious/depressed (Boomsma et al. 2005), attention problems (Rietveld et al. 2003), withdrawn behavior (Hoekstra et al. 2008), aggression (Hudziak et al. 2003), thought problems (Abdellaoui et al. 2008), internalizing and externalizing domains (Bartels et al. 2004, 2003; Bartels et al. 2007b). The larger collection of NTR data in the current paper allowed for analysis of categorical data under a threshold model. Several new scales were analyzed for the first time using multiple rater assessments at age 7, such as somatic complaints, rule-breaking behavior, social problems, dysregulation profile and total problems score. In addition, the earlier papers had focus on separate scales and domains, whereas all CBCL scales were explored simultaneously in the current study, allowing for comparison between scales. Our results showed that heritability estimates of internalizing, externalizing, dysregulation profile and total problems score in comparison to subscales comprising them, varies. The estimates of the unique aspect of the child behavior rated by mother are more variable across the internalizing scale subscales, than they are for father. This trend is not reflected in the internalizing scale, where all three phenotypes are combined. In addition, the absence of genetic effects estimated for unique aspect of maternal rating of the child’s behavior is likely driven by the anxious/depressed scale and not by others. In contrast, for the externalizing scale estimates of the contribution of genetic and environmental components to the variation of the phenotype shared by both parents were more variable across subscales. Only for the dysregulation profile and total problems scales a specific paternal contribution was accounted for by rater bias reflected by a significant Vc, unique component. A possible explanation is the heterogeneity of these measures in comparison to homogeneous single scales. We did not observe any sex differences in genetic architecture or in parental agreement for behavior rated for girls and boys, except for aggressive behavior, externalizing and the dysregulation profile, but observed the well-known differences between boys and girls for mean scores. Also, we observed that mothers rated the behavioral and emotional problems in their offspring higher than fathers.

Results obtained in our study are in line with earlier studies of CBCL 6–18 scales in twins aged 7. Both studies of single or multiple raters reported genetic influence on variability in behavioral and emotional problems (Brendgen et al. 2005; Eley et al. 1999; Haberstick et al. 2006; Hudziak et al. 2000; Spatola et al. 2007). In Brendgen et al. (2005), peers’ and teachers’ assessments were used to study genetic influences on social and physical aggression in 6 year olds, and heritability estimates were similar in magnitude between raters. The phenotypic correlation between teachers and mother ratings of aggressive behavior was moderate (*r* = 0.20) in a study of Haberstick et al. (2006) and heritability estimates differed in magnitude between raters for children at age 7 and the authors suggested that parents and teacher provide unique information that can be specific to the settings. In a study by Eley et al. (1999), sex-differences in aggressive antisocial behavior were reported for boys and girls, which were also detected in our study. To our knowledge there is limited research on somatic complaints, rule-breaking behavior, social problems scales and the dysregulation profile of CBCL 6–18 at this specific age. For the latter, the agreement and disagreement between raters were reported in an American non-twin cohort (Althoff et al. 2010). A report based on an Italian sample of twins (N = 398 pairs), rated by mothers, in age range from 8 to 17 years, showed no additive genetic effect, but 54% of common and 46% of unique environment effects for social problems (Spatola et al. 2007). Because heritability might change as a function of age (Bergen et al. 2007; Polderman et al. 2015), previous reports on younger and older twins are not directly comparable to the current study. These findings have implications for molecular genetic studies.

### Implications of our findings for molecular genetic studies

In molecular genetic studies, the distinction between rater bias and rater specific assessment of child’s behavior may have implications for the estimation of the SNP-heritability of behavioral and emotional problems. GWAS and GREML analyses will benefit from the determination to what extent two different sources of disagreement contribute to the phenotypic variance and affect the covariance. For example, differences in mother and father ratings suggest using rater as a covariate, if raters information is combined. In the recent study of Pappa et al. (2015) SNP-heritability of a range of children’s behavior problems were estimated. Attention deficit hyperactivity disorder (ADHD) related scales and externalizing behavior were assessed by both mother and teacher. Estimates of SNP-heritability of attention problems for teacher’s ratings and of ADHD Combined scale for Conner’s Parent Rating scale were 0.71 (S.E. = 0.22, n = 1495, *p* value < 0.001) and 0.40 (S.E. = 0.14, n = 2262, p < 0.01) respectively. For externalizing behavior scale estimates of SNP-heritability were 0.44 (S.E. = 0.22, n = 1495, p < 0.05) for teacher’s ratings and 0.12 (S.E. = 0.10, n = 3174, p = 0.13) for maternal ratings. The differences in SNP-heritability estimates are consistent with the findings obtained from twin studies, which account for rater specific effects. As was suggested in a study of attention problems by (Derks et al. 2006) both mother and teacher provide valid, but specific information about a child’s behavior in addition to a commonly assessed part. Therefore, variation explained by SNPs in teachers and mothers ratings may be represented by different, partly overlapping, genetic loci.

Our investigation of rater common and rater specific contributions to phenotypic variation serves as an indication of whether or not combined analyses of different informant ratings are likely to be fruitful. The substantial genetic correlations between different raters as evident from our results, suggest two practical guidelines: when studies have collected data from either fathers or mothers, the shared genetic aetiology in parental ratings indicates that is possible to analyze paternal and maternal assessments in a single GWA study or meta-analysis. Secondly, if a study has collected information from both parents, a gain in statistical power should be realized in a GWA study by simultaneous analysis of the data.

The power of various ways of modeling bivariate phenotype information, including analyses based on sum and factor scores, exploratory factor analysis (EFA), MANOVA, and combined multivariate analyses (CMV) were explored by Medland and Neale (2010), Minica et al. (2010), Van Der Sluis et al. (2010). Each of these approaches was evaluated in terms of power to discover genetic loci. Based on results of these studies, if the genetic correlation between different raters is very high, implying that genetic loci, that influence parental ratings, overlap almost completely, combining the ratings in a single trait, using sum score is justifiable (Minica et al. 2010). If the correlations are moderate to high, one might prefer a technique that has high power when loci are expected to influence the shared, as well as unique part of the phenotype as assessed by the different raters (Medland and Neale 2010). Finally, if the genetic correlations between raters is low to moderate, one might prefer to perform separate analysis in either rater and combine the resulting p-values by using trait-based association test that uses extended simes procedure (TATES) (van der Sluis et al. 2013).

In current study we considered parental ratings and did not make an attempt to analyze rater effects based on teachers ratings or on self-assessments of children. Inclusion of other raters will convey additional information about possible combined or separate analysis of problem behaviors assessed by multiple raters.

Based on the results reported in this paper, we conclude that aggregating multiple raters’ in genetic studies of childhood psychopathology potentially will improve power. At age 7, our study showed that heritability of phenotypes reflecting a shared perspective on the child’s problem behavior is substantially higher than that of unique view. These results suggest a model in which genome wide analysis of different raters are combined into a single trait, accounting for genetic correlation, and differences in heritability, could prove optimal. For traits with a (somewhat) lower genetic correlation or if including further raters, for which substantial rater specific genetic effects are present (e.g. self ratings, teacher ratings, clinician ratings), a multitude of multivariate genetic analysis tools exist.

## Electronic supplementary material

Below is the link to the electronic supplementary material.

Supplementary material 1 (DOCX 103 kb)
