# Supplementary material for: Heritability of Behavioral Problems in 7-Year Olds Based on Shared and Unique Aspects of Parental Views
Source: Behav Genet. 2016 Oct 28;47(2):152–63. doi: 10.1007/s10519-016-9823-1 (PMC5306273; doi:10.1007/s10519-016-9823-1)
Supplement: Supplementary file 1 — Supplementary material 1 (DOCX 103 kb) [file 10519_2016_9823_MOESM1_ESM.docx]

**Heritability of behavioral problems in 7-year olds based on shared and unique aspects of parental views**

**Supplementary Material**

Iryna O. Fedko^1,*^, Laura W. Wesseldijk^1,2^, Michel G Nivard^1^, Jouke-Jan Hottenga^1,2^, Catharina E.M. van Beijsterveldt^1^, Christel M. Middeldorp^1,3,4^, Meike Bartels^1,2,3^, and Dorret I. Boomsma^1,2,3^

^1^Department of Biological Psychology, Vrije Universiteit Amsterdam, The Netherlands

^2^EMGO^+^ institute for Health and Care Research, VU Medical Center, Amsterdam, The Netherlands

^3^Neuroscience Campus Amsterdam, Amsterdam, The Netherlands

^4^GGZ inGeest / VU Medical Center, Department of Child and Adolescent Psychiatry, Amsterdam, The Netherlands

* Corresponding author: Iryna O. Fedko, Department of Biological Psychology, VU University Amsterdam, Van der Boechorststraat 1, 1081BT, Amsterdam, The Netherlands, e-mail: [i.o.fedko@vu.nl](mailto:i.o.fedko@vu.nl), Tel: +31-20-598-34-25, Fax: +31-20-598-88-32.

Running title: Heritability of behavioral problems in 7-year olds

**Supplementary Table 1.** Descriptive statistics of raw CBCL 6-18 scales.

|  | Mother | | | | Father | | | |
| --- | --- | --- | --- | --- | --- | --- | --- | --- |
|  | N | Mean (SD) | Skewness (SE) | Kurtosis (SE) | N | Mean (SD) | Skewness (SE) | Kurtosis (SE) |
| Anxious/depressed | 24031 | 2.25 (2.58) | 1.91 (0.02) | 5.07 (0.03) | 17042 | 1.73 (2.15) | 2.10 (0.02) | 6.53 (0.04) |
| Withdrawn/depressed | 24001 | 1.14 (1.60) | 2.26 (0.02) | 6.73 (0.03) | 16988 | 0.92 (1.44) | 2.48 (0.02) | 8.11 (0.04) |
| Somatic complaints | 23794 | 1.20 (1.65) | 2.23 (0.02) | 8.11 (0.03) | 16931 | 0.87 (1.34) | 2.48 (0.02) | 10.10 (0.04) |
| Rule-breaking behavior | 24028 | 1.33 (1.83) | 2.06 (0.02) | 5.88 (0.03) | 16999 | 1.16 (1.69) | 2.30 (0.02) | 8.27 (0.04) |
| Aggressive behavior | 24006 | 5.10 (4.89) | 1.45 (0.02) | 2.60 (0.03) | 16995 | 4.40 (4.40) | 1.45 (0.02) | 2.55 (0.04) |
| Social problems | 24017 | 2.08 (2.39) | 1.88 (0.02) | 4.78 (0.03) | 17028 | 1.72 (2.11) | 2.03 (0.02) | 5.83 (0.04) |
| Thought problems | 23898 | 1.49 (1.98) | 2.28 (0.02) | 7.52 (0.03) | 16938 | 1.11 (1.66) | 2.66 (0.02) | 11.16 (0.04) |
| Attention problems | 24041 | 3.04 (3.03) | 1.25 (0.02) | 1.69 (0.03) | 17028 | 2.69 (2.81) | 1.30 (0.02) | 1.93 (0.04) |
| Internalizing | 23599 | 4.58 (4.61) | 1.87 (0.02) | 5.05 (0.03) | 16804 | 3.51 (3.84) | 2.12 (0.02) | 7.11 (0.04) |
| Externalizing | 23962 | 6.43 (6.26) | 1.58 (0.02) | 3.30 (0.03) | 16950 | 5.56 (5.64) | 1.61 (0.02) | 3.46 (0.04) |
| Dysregulation profile | 23841 | 10.39 (8.59) | 1.38 (0.02) | 2.60 (0.03) | 16908 | 8.81 (7.68) | 1.43 (0.02) | 2.69 (0.04) |
| Total problems | 23936 | 20.92 (16.45) | 1.51 (0.02) | 3.30 (0.03) | 16957 | 17.26 (14.50) | 1.67 (0.02) | 4.50 (0.04) |

**Supplementary Table 2.** Relationship between the categories and raw CBCL 6-18 scores. The raw maternal and paternal scores for each scale (rows) were categorized into three categories. Ranges of raw scores in each category are shown in ‘min’ and ‘max’ columns.

|  | Mother | | | | | | Father | | | | | |
| --- | --- | --- | --- | --- | --- | --- | --- | --- | --- | --- | --- | --- |
|  | Category 1 | | Category 2 | | Category 3 | | Category 1 | | Category 2 | | Category 3 | |
|  | min | max | min | max | min | max | min | max | min | max | min | max |
| Anxious/depressed | 0 | 1 | 2 | 2 | 3 | 25 | 0 | 1 | 2 | 2 | 3 | 21 |
| Withdrawn/depressed | 0 | 0 | 1 | 1 | 2 | 15 | 0 | 0 | 1 | 1 | 2 | 13 |
| Somatic complaints | 0 | 0 | 1 | 1 | 2 | 22 | 0 | 0 | 1 | 1 | 2 | 18 |
| Rule-breaking behavior | 0 | 0 | 1 | 1 | 2 | 17 | 0 | 0 | 1 | 1 | 2 | 20 |
| Aggressive behavior | 0 | 2 | 3 | 6 | 7 | 35 | 0 | 2 | 3 | 6 | 7 | 30 |
| Social problems | 0 | 1 | 2 | 2 | 3 | 20 | 0 | 1 | 2 | 2 | 3 | 19 |
| Thought problems | 0 | 0 | 1 | 1 | 2 | 19 | 0 | 0 | 1 | 1 | 2 | 18 |
| Attention problems | 0 | 1 | 2 | 4 | 5 | 20 | 0 | 1 | 2 | 4 | 5 | 19 |
| Internalizing | 0 | 2 | 3 | 5 | 6 | 45 | 0 | 2 | 3 | 5 | 6 | 40 |
| Externalizing | 0 | 2 | 3 | 7 | 8 | 48 | 0 | 2 | 3 | 7 | 8 | 43 |
| Dysregulation profile | 0 | 5 | 6 | 12 | 13 | 70 | 0 | 5 | 6 | 12 | 13 | 63 |
| Total problems | 0 | 11 | 12 | 22 | 23 | 143 | 0 | 11 | 12 | 22 | 23 | 142 |

**Supplementary Table 3.** Models fitting to CBCL 6-18 empirical scales: 4x4 zygosity by sex correlations matrix and 8 thresholds with submodels, psychometric model with submodels.

| CBCL 6-18 scale |  | Model | Estimated parameters | -2 LL | df | Compared to model | Δ LL | Δ df | P |
| --- | --- | --- | --- | --- | --- | --- | --- | --- | --- |
| Anxious/depressed | 1 | Saturated model | 27 | 72112.62 | 41046 | - | - | - | - |
|  | 2 | *Equal thresholds boys and girls* | 23 | 72150.41 | 41050 | 1 | 37.79 | 4 | 1.2×10^-7^ |
|  | 3 | *Parental agreement across zygosity* | 25 | 72114.75 | 41048 | 1 | 2.12 | 2 | 0.35 |
|  | 4 | *Parental agreement across sex* | 24 | 72115.01 | 41049 | 3 | 0.26 | 1 | 0.61 |
|  | 5 | *MZM=MZF & DZM = DZF* | 18 | 72121.16 | 41055 | 4 | 6.15 | 6 | 0.41 |
|  | 6 | *DZ=DOS* | 15 | 72128.16 | 41058 | 5 | 7.00 | 3 | 0.07 |
|  | 7 | Psychometric model | 17 | 72128.16 | 41058 | - | - | - | - |
|  | 8 | *no C* | 16 | 72129.44 | 41059 | 7 | 1.28 | 1 | 0.26 |
|  | 9 | *no Am* | 15 | 72136.16 | 41060 | 8 | 6.72 | 1 | 0.01 |
|  | 10 | *no Af* | 15 | 72149.50 | 41060 | 8 | 20.06 | 1 | 7.5×10^-6^ |
|  | 11 | *no Am, no Af* | 14 | 72164.78 | 41061 | 8 | 35.34 | 2 | 2.1×10^-8^ |
| Withdrawn/depressed | 1 | Saturated model | 27 | 77275.25 | 40962 | - | - | - | - |
|  | 2 | *Equal thresholds boys and girls* | 23 | 77289.28 | 40966 | 1 | 14.03 | 4 | 0.01 |
|  | 3 | *Parental agreement across zygosity* | 25 | 77275.45 | 40964 | 1 | 0.20 | 2 | 0.91 |
|  | 4 | *Parental agreement across sex* | 24 | 77275.49 | 40965 | 3 | 0.05 | 1 | 0.83 |
|  | 5 | *MZM=MZF & DZM = DZF* | 18 | 77291.10 | 40971 | 4 | 15.61 | 6 | 0.02 |
|  | 6 | *DZ=DOS* | 15 | 77300.52 | 40974 | 5 | 9.42 | 3 | 0.02 |
|  | 7 | Psychometric model | 17 | 77316.88 | 40974 | - | - | - | - |
|  | 8 | *no C* | 16 | 77316.88 | 40975 | 7 | 0.00 | 1 | 1.00 |
|  | 9 | *no Am* | 15 | 77367.63 | 40976 | 8 | 50.75 | 1 | 1.0×10^-12^ |
|  | 10 | *no Af* | 15 | 77342.44 | 40976 | 8 | 25.56 | 1 | 4.3×10^-7^ |
|  | 11 | *no Am, no Af* | 14 | 77414.57 | 40977 | 8 | 97.69 | 2 | 6.1×10^-22^ |

**Supplementary Table 3.** Continued.

| CBCL 6-18 scale |  | Model | Estimated parameters | -2 LL | df | Compared to model | Δ LL | Δ df | P |
| --- | --- | --- | --- | --- | --- | --- | --- | --- | --- |
| Somatic complaints | 1 | Saturated model | 27 | 75333.91 | 40698 | - | - | - | - |
|  | 2 | *Equal thresholds boys and girls* | 23 | 75388.41 | 40702 | 1 | 54.50 | 4 | 4.1×10^-11^ |
|  | 3 | *Parental agreement across zygosity* | 25 | 75336.10 | 40700 | 1 | 2.19 | 2 | 0.34 |
|  | 4 | *Parental agreement across sex* | 24 | 75336.19 | 40701 | 3 | 0.09 | 1 | 0.77 |
|  | 5 | *MZM=MZF & DZM = DZF* | 18 | 75340.12 | 40707 | 4 | 3.93 | 6 | 0.69 |
|  | 6 | *DZ=DOS* | 15 | 75341.72 | 40710 | 5 | 1.60 | 3 | 0.66 |
|  | 7 | Psychometric model | 17 | 75341.56 | 40710 | - | - | - | - |
|  | 8 | *no C* | 16 | 75344.69 | 40711 | 7 | 3.13 | 1 | 0.08 |
|  | 9 | *no Am* | 15 | 75355.51 | 40712 | 8 | 10.82 | 1 | 1.0×10^-3^ |
|  | 10 | *no Af* | 15 | 75372.68 | 40712 | 8 | 27.99 | 1 | 1.2×10^-7^ |
|  | 11 | *no Am, no Af* | 14 | 75398.35 | 40713 | 8 | 53.66 | 2 | 2.2×10^-12^ |
| Rule-breaking | 1 | Saturated model | 27 | 72083.57 | 41000 | - | - | - | - |
| behavior | 2 | *Equal thresholds boys and girls* | 23 | 72442.12 | 41004 | 1 | 358.55 | 4 | 2.5×10^-76^ |
|  | 3 | *Parental agreement across zygosity* | 25 | 72084.30 | 41002 | 1 | 0.74 | 2 | 0.69 |
|  | 4 | *Parental agreement across sex* | 24 | 72090.07 | 41003 | 3 | 5.77 | 1 | 0.02 |
|  | 5 | *MZM=MZF & DZM = DZF* | 18 | 72099.72 | 41009 | 4 | 9.64 | 6 | 0.14 |
|  | 6 | *DZ=DOS* | 15 | 72104.29 | 41012 | 5 | 4.57 | 3 | 0.21 |
|  | 7 | Psychometric model | 17 | 72104.29 | 41012 | - | - | - | - |
|  | 8 | *no C* | 16 | 72289.73 | 41013 | 7 | 185.44 | 1 | 3.1×10^-42^ |
|  | 9 | *no Am* | 16 | 72142.36 | 41013 | 7 | 38.07 | 1 | 6.8×10^-10^ |
|  | 10 | *no Af* | 16 | 72135.86 | 41013 | 7 | 31.57 | 1 | 1.9×10^-8^ |
|  | 11 | *no Am, no Af* | 15 | 72185.32 | 41014 | 7 | 81.03 | 2 | 2.5×10^-18^ |

**Supplementary Table 3.** Continued.

| CBCL 6-18 scale |  | Model | Estimated parameters | -2 LL | df | Compared to model | Δ LL | Δ df | P |
| --- | --- | --- | --- | --- | --- | --- | --- | --- | --- |
| Aggressive behavior | 1 | Saturated model | 27 | 73373.97 | 40974 | - | - | - | - |
|  | 2 | *Equal thresholds boys and girls* | 23 | 73754.93 | 40978 | 1 | 380.96 | 4 | 3.6×10^-81^ |
|  | 3 | *Parental agreement across zygosity* | 25 | 73374.20 | 40976 | 1 | 0.22 | 2 | 0.89 |
|  | 4 | *Parental agreement across sex* | 24 | 73390.07 | 40977 | 3 | 15.88 | 1 | 6.8×10^-5^ |
|  | 5 | *MZM=MZF & DZM = DZF* | 18 | 73395.31 | 40983 | 4 | 5.24 | 6 | 0.51 |
|  | 6 | *DZ=DOS* | 15 | 73400.25 | 40986 | 5 | 4.94 | 3 | 0.18 |
|  | 7 | Psychometric model | 17 | 73400.25 | 40986 | - | - | - | - |
|  | 8 | *no C* | 16 | 73463.21 | 40987 | 7 | 62.96 | 1 | 2.1×10^-15^ |
|  | 9 | *no Am* | 16 | 73424.82 | 40987 | 7 | 24.57 | 1 | 7.2×10^-7^ |
|  | 10 | *no Af* | 16 | 73416.47 | 40987 | 7 | 16.22 | 1 | 5.6×10^-5^ |
|  | 11 | *no Am, no Af* | 15 | 73454.24 | 40988 | 7 | 53.99 | 2 | 1.9×10^-12^ |
| Social problems | 1 | Saturated model | 27 | 70539.94 | 41018 | - | - | - | - |
|  | 2 | *Equal thresholds boys and girls* | 23 | 70605.89 | 41022 | 1 | 65.95 | 4 | 1.6×10^-13^ |
|  | 3 | *Parental agreement across zygosity* | 25 | 70541.34 | 41020 | 1 | 1.40 | 2 | 0.50 |
|  | 4 | *Parental agreement across sex* | 24 | 70546.81 | 41021 | 3 | 5.46 | 1 | 0.02 |
|  | 5 | *MZM=MZF & DZM = DZF* | 18 | 70548.85 | 41027 | 4 | 2.05 | 6 | 0.92 |
|  | 6 | *DZ=DOS* | 15 | 70551.79 | 41030 | 5 | 2.94 | 3 | 0.40 |
|  | 7 | Psychometric model | 17 | 70551.79 | 41030 | - | - | - | - |
|  | 8 | *no C* | 16 | 70565.87 | 41031 | 7 | 14.07 | 1 | 1.8×10^-4^ |
|  | 9 | *no Am* | 16 | 70580.23 | 41031 | 7 | 28.43 | 1 | 9.7×10^-8^ |
|  | 10 | *no Af* | 16 | 70574.55 | 41031 | 7 | 22.76 | 1 | 1.8×10^-6^ |
|  | 11 | *no Am, no Af* | 15 | 70615.59 | 41032 | 7 | 63.79 | 2 | 1.4×10^-14^ |

**Supplementary Table 3.** Continued.

| CBCL 6-18 scale |  | Model | Estimated parameters | -2 LL | df | Compared to model | Δ LL | Δ df | P |
| --- | --- | --- | --- | --- | --- | --- | --- | --- | --- |
| Thought problems | 1 | Saturated model | 27 | 76500.53 | 40809 | - | - | - | - |
|  | 2 | *Equal thresholds boys and girls* | 23 | 76792.95 | 40813 | 1 | 292.42 | 4 | 4.7×10^-62^ |
|  | 3 | *Parental agreement across zygosity* | 25 | 76501.05 | 40811 | 1 | 0.52 | 2 | 0.77 |
|  | 4 | *Parental agreement across sex* | 24 | 76501.24 | 40812 | 3 | 0.18 | 1 | 0.67 |
|  | 5 | *MZM=MZF & DZM = DZF* | 18 | 76508.16 | 40818 | 4 | 6.93 | 6 | 0.33 |
|  | 6 | *DZ=DOS* | 15 | 76512.26 | 40821 | 5 | 4.09 | 3 | 0.25 |
|  | 7 | Psychometric model | 17 | 76512.26 | 40821 | - | - | - | - |
|  | 8 | *no C* | 16 | 76525.54 | 40822 | 7 | 13.28 | 1 | 2.7×10^-4^ |
|  | 9 | *no Am* | 16 | 76559.62 | 40822 | 7 | 47.37 | 1 | 5.9×10^-12^ |
|  | 10 | *no Af* | 16 | 76578.25 | 40822 | 7 | 66.00 | 1 | 4.5×10^-16^ |
|  | 11 | *no Am, no Af* | 15 | 76640.10 | 40823 | 7 | 127.84 | 2 | 1.7×10^-28^ |
| Attention problems | 1 | Saturated model | 27 | 74865.33 | 41042 | - | - | - | - |
|  | 2 | *Equal thresholds boys and girls* | 23 | 75444.63 | 41046 | 1 | 579.30 | 4 | 4.7×10^-124^ |
|  | 3 | *Parental agreement across zygosity* | 25 | 74865.62 | 41044 | 1 | 0.29 | 2 | 0.87 |
|  | 4 | *Parental agreement across sex* | 24 | 74866.87 | 41045 | 3 | 1.25 | 1 | 0.26 |
|  | 5 | *MZM=MZF & DZM = DZF* | 18 | 74878.10 | 41051 | 4 | 11.23 | 6 | 0.08 |
|  | 6 | *DZ=DOS* | 15 | 74882.93 | 41054 | 5 | 4.83 | 3 | 0.18 |
|  | 7 | Psychometric model | 17 | 74910.91 | 41054 | - | - | - | - |
|  | 8 | *no Dm* | 16 | 74910.90 | 41055 | 7 | -0.01 | 1 | 1.00 |
|  | 9 | *no Df* | 15 | 74910.90 | 41056 | 8 | 0.00 | 1 | 1.00 |
|  | 10 | *no Am* | 14 | 75471.23 | 41057 | 9 | 560.33 | 1 | 7.1×10^-124^ |
|  | 11 | *no Af* | 14 | 75545.44 | 41057 | 9 | 634.54 | 1 | 5.1×10^-140^ |
|  | 12 | *no Am, Af* | 13 | 76191.07 | 41058 | 9 | 1280.17 | 2 | 1.0×10^-278^ |

**Supplementary Table 3.** Continued.

| CBCL 6-18 scale |  | Model | Estimated parameters | -2 LL | df | Compared to model | Δ LL | Δ df | P |
| --- | --- | --- | --- | --- | --- | --- | --- | --- | --- |
| Internalizing | 1 | Saturated model | 27 | 75160.06 | 40376 | - | - | - | - |
|  | 2 | *Equal thresholds boys and girls* | 23 | 75187.11 | 40380 | 1 | 27.05 | 4 | 1.9×10^-5^ |
|  | 3 | *Parental agreement across zygosity* | 25 | 75162.65 | 40378 | 1 | 2.59 | 2 | 0.27 |
|  | 4 | *Parental agreement across sex* | 24 | 75169.23 | 40379 | 3 | 6.58 | 1 | 0.01 |
|  | 5 | *MZM=MZF & DZM = DZF* | 18 | 75181.43 | 40385 | 4 | 12.20 | 6 | 0.06 |
|  | 6 | *DZ=DOS* | 15 | 75186.48 | 40388 | 5 | 5.05 | 3 | 0.17 |
|  | 7 | Psychometric model | 17 | 75187.24 | 40388 | - | - | - | - |
|  | 8 | *no C* | 16 | 75221.28 | 40389 | 7 | 34.04 | 1 | 5.4×10^-9^ |
|  | 9 | *no Am* | 16 | 75189.21 | 40389 | 7 | 1.97 | 1 | 0.16 |
|  | 10 | *no Af* | 16 | 75198.34 | 40389 | 7 | 11.10 | 1 | 8.6×10^-4^ |
|  | 11 | *no Am, no Af* | 15 | 75202.25 | 40390 | 7 | 15.00 | 2 | 5.5×10^-4^ |
| Externalizing | 1 | Saturated model | 27 | 72875.88 | 40885 | - | - | - | - |
|  | 2 | *Equal thresholds boys and girls* | 23 | 73296.23 | 40889 | 1 | 420.34 | 4 | 1.1×10^-89^ |
|  | 3 | *Parental agreement across zygosity* | 25 | 72876.47 | 40887 | 1 | 0.58 | 2 | 0.75 |
|  | 4 | *Parental agreement across sex* | 24 | 72892.77 | 40888 | 3 | 16.30 | 1 | 5.4×10^-5^ |
|  | 5 | *MZM=MZF & DZM = DZF* | 18 | 72899.02 | 40894 | 4 | 6.25 | 6 | 0.40 |
|  | 6 | *DZ=DOS* | 15 | 72904.88 | 40897 | 5 | 5.86 | 3 | 0.12 |
|  | 7 | Psychometric model | 17 | 72905.00 | 40897 | - | - | - | - |
|  | 8 | *no C* | 16 | 73038.14 | 40898 | 7 | 133.14 | 1 | 8.4×10^-31^ |
|  | 9 | *no Am* | 16 | 72951.51 | 40898 | 7 | 46.51 | 1 | 9.1×10^-12^ |
|  | 10 | *no Af* | 16 | 72917.44 | 40898 | 7 | 12.44 | 1 | 4.2×10^-4^ |
|  | 11 | *no Am, no Af* | 15 | 72977.68 | 40899 | 7 | 72.68 | 2 | 1.6×10^-16^ |

**Supplementary Table 3.** Continued.

| CBCL 6-18 scale |  | Model | Estimated parameters | -2 LL | df | Compared to model | Δ LL | Δ df | P |
| --- | --- | --- | --- | --- | --- | --- | --- | --- | --- |
| Dysregulation profile | 1 | Saturated model | 27 | 72916.78 | 40722 | - | - | - | - |
|  | 2 | *Equal thresholds boys and girls* | 23 | 73345.75 | 40726 | 1 | 428.97 | 4 | 1.5×10^-91^ |
|  | 3 | *Parental agreement across zygosity* | 25 | 72919.22 | 40724 | 1 | 2.44 | 2 | 0.30 |
|  | 4 | *Parental agreement across sex* | 24 | 72945.21 | 40725 | 3 | 25.98 | 1 | 3.4×10^-7^ |
|  | 5 | *MZM=MZF & DZM = DZF* | 18 | 72948.13 | 40731 | 4 | 2.93 | 6 | 0.82 |
|  | 6 | *DZ=DOS* | 15 | 72952.54 | 40734 | 5 | 4.41 | 3 | 0.22 |
|  | 7 | Psychometric model | 17 | 72952.54 | 40734 | - | - | - | - |
|  | 8 | *no C* | 16 | 73051.38 | 40735 | 7 | 98.84 | 1 | 2.7×10^-23^ |
|  | 9 | *no Am* | 16 | 72987.24 | 40735 | 7 | 34.70 | 1 | 3.8×10^-9^ |
|  | 10 | *no Af* | 16 | 72958.13 | 40735 | 7 | 5.59 | 1 | 0.02 |
|  | 11 | *no Am, no Af* | 15 | 72999.75 | 40736 | 7 | 47.21 | 2 | 5.6×10^-11^ |
| Total problems | 1 | Saturated model | 27 | 71530.16 | 40866 | - | - | - | - |
|  | 2 | *Equal thresholds boys and girls* | 23 | 71929.17 | 40870 | 1 | 399.01 | 4 | 4.6×10^-85^ |
|  | 3 | *Parental agreement across zygosity* | 25 | 71530.17 | 40868 | 1 | 0.00 | 2 | 1.00 |
|  | 4 | *Parental agreement across sex* | 24 | 71537.02 | 40869 | 3 | 6.85 | 1 | 0.01 |
|  | 5 | *MZM=MZF & DZM = DZF* | 18 | 71545.51 | 40875 | 4 | 8.49 | 6 | 0.20 |
|  | 6 | *DZ=DOS* | 15 | 71551.16 | 40878 | 5 | 5.65 | 3 | 0.13 |
|  | 7 | Psychometric model | 17 | 71551.16 | 40878 | - | - | - | - |
|  | 8 | *no C* | 16 | 71851.20 | 40879 | 7 | 300.04 | 1 | 3.2×10^-67^ |
|  | 9 | *no Am* | 16 | 71573.65 | 40879 | 7 | 22.50 | 1 | 2.1×10^-6^ |
|  | 10 | *no Af* | 16 | 71553.24 | 40879 | 7 | 2.08 | 1 | 0.15 |
|  | 11 | *no Am, no Af* | 15 | 71578.84 | 40880 | 7 | 27.68 | 2 | 9.8×10^-7^ |

df = degrees of freedom, -2LL = - 2 log likelihood, C = shared environmental variance of the common part of total variance, Am = additive genetic variance of mother specific component of total variance, Af = additive genetic variance of father specific component of total variance, Dm = dominant genetic variance of mother specific component of total variance, Df = dominant genetic variance of father specific component of total variance, MZM = monozygotic twins males, MZF = monozygotic twins females, DZM = dizygotic twins males, DZF = dizygotic twins females, DZ – same sex dizygotic twins, DOS – opposite sex dizygotic twins.
